# Long-Term Overall Survival After Selective Internal Radiation Therapy for Locally Advanced Hepatocellular Carcinomas: Updated Analysis of DOSISPHERE-01 Trial

**DOI:** 10.2967/jnumed.123.266211

**Published:** 2024-02

**Authors:** Etienne Garin, Lambros Tselikas, Boris Guiu, Julia Chalaye, Yan Rolland, Thierry de Baere, Eric Assenat, Vania Tacher, Xavier Palard, Desirée Déandreis, Denis Mariano-Goulart, Giuliana Amaddeo, Karim Boudjema, Antoine Hollebecque, Mohamad Azhar Meerun, Helen Regnault, Eric Vibert, Boris Campillo-Gimenez, Julien Edeline

**Affiliations:** 1 Cancer Institute Eugene Marquis, Rennes, France;; 2 University of Rennes, INSERM, INRAE, Nutrition Métabolismes et Cancer U1317, Rennes, France;; 3 Gustave Roussy, University of Paris-Saclay, Villejuif, France;; 4 Montpellier University Hospital, Montpellier, France;; 5 AP-HP, Hopitaux Universitaires Henri Mondor, Creteil, France;; 6 University of Rennes, INSERM, LTSI–UMR 1099, Rennes, France;; 7 Department of Hepatobiliary and Digestive Surgery, CHU Rennes, Rennes, France;; 8 Centre Hepato-Biliaire, Paul Brousse Hospital, AP-HP, Paris Saclay University, Villejuif, France; and; 9 University of Rennes, INSERM, COSS–UMR_S 1242, Rennes, France

**Keywords:** hepatocellular carcinoma, ^90^Y-loaded glass microspheres, personalized dosimetry, downstaged, portal vein thrombosis

## Abstract

Interim analysis of the DOSISPHERE-01 study demonstrated a strong improvement in response and overall survival (OS) on using ^90^Y-loaded glass microspheres with personalized dosimetry compared with standard dosimetry in patients with nonoperable locally advanced hepatocellular carcinoma. This report sought to provide a long-term analysis of OS. **Methods:** In this phase II study (ClinicalTrials.gov identifier NCT02582034), treatment was randomly assigned (1:1) with the goal to deliver either at least 205 Gy (if possible >250–300 Gy) to the index lesion in the personalized dosimetry approach (PDA) or 120 ± 20 Gy to the treated volume in the standard dosimetry approach (SDA). The 3-mo response of the index lesion was the primary endpoint, with OS being one of the secondary endpoints. This report is a post hoc long-term analysis of OS. **Results:** Overall, 60 hepatocellular carcinoma patients with at least 1 lesion larger than 7 cm and more than 30% of hepatic reserve were randomized (intent-to-treat population: PDA, *n* = 31; SDA, *n* = 29), with 56 actually treated (modified intent-to-treat population: *n* = 28 in each arm). The median follow-up for long-term analysis was 65.8 mo (range, 2.1–73.1 mo). Median OS was 24.8 mo and 10.7 mo (hazard ratio [HR], 0.51; 95% CI, 0.29–0.9; *P* = 0.02) for PDA and SDA, respectively, in the modified intent-to-treat population. Median OS was 22.9 mo for patients with a tumor dose of at least 205 Gy, versus 10.3 mo for those with a tumor dose of less than 205 Gy (HR, 0.42; 95% CI, 0.22–0.81; *P* = 0.0095), and was 22.9 mo for patients with a perfused liver dose of 150 Gy or higher, versus 10.3 mo for those with a perfused liver dose of less than 150 Gy (HR, 0.42; 95% CI, 0.23–0.75; *P* = 0.0033). Lastly, median OS was not reached in patients who were secondarily resected (*n* = 11, 10 in the PDA group and 1 in the SDA group), versus 10.8 mo in those without secondary resection (*n* = 45) (HR, 0.17; 95% CI, 0.065–0.43; *P* = 0.0002). Only resected patients displayed favorable long-term OS rates, meaning an OS of more than 50% at 5 y. **Conclusion:** After longer follow-up, personalized dosimetry sustained a meaningful improvement in OS, which was dramatically improved for patients who were accurately downstaged toward resection, including most portal vein thrombosis patients.

## Visual Abstract

**Figure:**
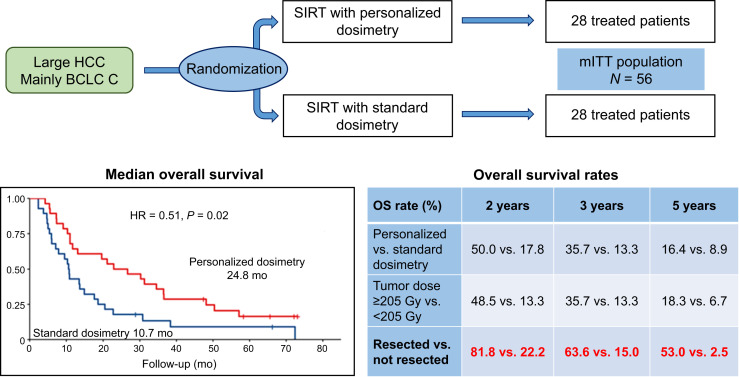

Hepatocellular carcinoma is the most common primary liver cancer, being the third leading cause of cancer-related death worldwide, with approximately 745,000 deaths reported annually (1). Selective internal radiation therapy (SIRT) using ^90^Y-loaded glass microspheres can be used for patients with early-stage to locally advanced hepatocellular carcinoma (2*,*^3^).

Despite the negativity of all randomized trials comparing ^90^Y-loaded resin microspheres versus sorafenib (4*–*6), without any personalized dosimetry used, the interest in SIRT for locally advanced hepatocellular carcinoma is returning because of the results of the randomized DOSISPHERE-01 study (7). This randomized phase II trial using ^90^Y-loaded microspheres sought to compare the effectiveness of ^90^Y-loaded microspheres using a personalized dosimetry approach (PDA) versus a standard dosimetry approach (SDA), which was stopped at the interim analysis because of the pronounced superiority of PDA in primary endpoint terms. Indeed, the 3-mo response of the index lesion was 71% in PDA versus only 36% in SDA (*P* = 0.0074) (7). On study analysis, median overall survival (OS) was significantly improved in the intent-to-treat population in the PDA group: 26.6 mo (95% CI, 11.7 mo to not reached) versus 10.7 mo (95% CI, 6.0–16.8 mo) in the SDA group (hazard ratio [HR], 0.42; 95% CI, 0.21–0.83; *P* = 0.0096) (7). However, at that time, OS evaluation was performed with a short follow-up time of 27.2 mo because of early trial termination at interim analysis. Moreover, a description of long-term follow-up of patients who could be resected after downsizing is important.

Our main objective here was to report the OS evaluation after updated longer-term follow-up in the DOSISPHERE-01 cohort.

## MATERIALS AND METHODS

### Study Design and Population

The study design and population have been published previously (7). Briefly, eligible patients were randomly assigned (1:1) to the PDA and SDA groups. Treatment was scheduled to deliver a tumor dose (TD) of at least 205 Gy (if possible >250–300 Gy) to the index lesion in the PDA group or 120 ± 20 Gy to the treated volume in the SDA group. The response rate of the index lesion at 3 mo, according to the criteria of the European Association for the Study of the Liver, was the primary endpoint, with OS being one of the secondary endpoints. Some of the most specific eligibility criteria applied in the DOSISPHERE-01 trial were at least 1 lesion larger than 7 cm; the ability to spare at least 30% of the liver volume from radiation; exclusion based on treatment simulation, including a high lung shunt leading to an excessive lung dose (providing >30 Gy); a digestive shunt; and poor tumor and/or poor portal vein thrombosis (PVT) targeting.

All patients provided written informed consent before undergoing study-specific procedures. The study was performed in accordance with the Declaration of Helsinki principles. The study protocol was approved by the ethics committee of the University Hospital La Cavalle Blanche (IRB-ID approval 2015-A00894-45) and registered on ClinicalTrials.gov (NCT02582034).

### Procedures

^90^Y-loaded glass microspheres were used with a lobar approach. The dosimetry evaluation was based on ^99m^Tc-macroaggregated albumin SPECT/CT (quantification as previously described (8)).

### Statistics

Results were presented according to the modified intent-to-treat population, defined as the overall treated patients. Long-term follow-up was estimated using the reverse Kaplan–Meier approach. OS curves were estimated by means of the Kaplan–Meier methodology and compared using log-rank tests. Product-limit estimates were presented by arm using median times and 1- to 5-y survival rates with the corresponding 2-sided 95% CI. HRs were computed using univariable Cox regression. A priori subgroup analyses were conducted as recorded in the initial DOSISPHERE protocol (7). Post hoc comparisons were added comparing survival curves according to TD (<205 Gy vs. ≥205 Gy), perfused liver dose (PLD) (<150 Gy vs. ≥150 Gy), and secondary resection (resected vs. not resected). A multivariable analysis of OS was also performed including these 3 additional variables and all previous subgroup factors. A Cox proportional-hazards model was fitted using significant variables (threshold < 0.15) from the univariate analysis. An ascending and descending stepwise procedure was used to select variables, minimizing the Akaike criteria. Data were analyzed using R version 4.2.1 (2022-06-23 ucrt; https://www.R-project.org/).

## RESULTS

The main individual characteristics of the study population are presented in Table 1. Median follow-up was 65.8 mo (range, 2.1–73.1 mo).

**TABLE 1.** Summary of Main Demographic and Baseline Characteristics of Patients in Modified Intent-to-Treat Population

Characteristic	PDA (*n* = 28)	SDA (*n* = 28)
Age (y)	64.8 ± 10.1	62.5 ± 63.7
Child classification		
A5	22 (78.6%)	22 (78.6%)
A6/B7	6 (21.4%)	6 (21.4%)
ECOG performance status		
0	16 (57.1%)	13 (46.4%)
1	12 (42.9%)	15 (53.6%)
BCLC classification		
B	3 (10.7%)	2 (7.1%)
C	25 (89.3%)	26 (92.9%)
Portal vein invasion		
Present	18 (64.3%)	21 (75%)
Absent	10 (35.7%)	7 (25%)
Cirrhosis etiology		
Alcohol	9 (32.1%)	9 (32.1%)
Viral hepatitis	7 (25%)	9 (32.1%)
Hemochromatosis	1 (3.6%)	0
NASH	3 (10.7%)	3 (10.7%)
Mixture (alcohol + other)	4 (14.3%)	3 (10.7%)
No cirrhosis	4 (14.3%)	4 (14.3%)
Tumor size (cm)	10.54 ± 2.43	10.92 ± 2.57

ECOG = Eastern Cooperative Oncology Group; BCLC = Barcelona Clinic of Liver Cancer; NASH = nonalcoholic steatohepatitis.

Qualitative data are number and percentage; continuous data are mean ± SD.

**FIGURE 1.**
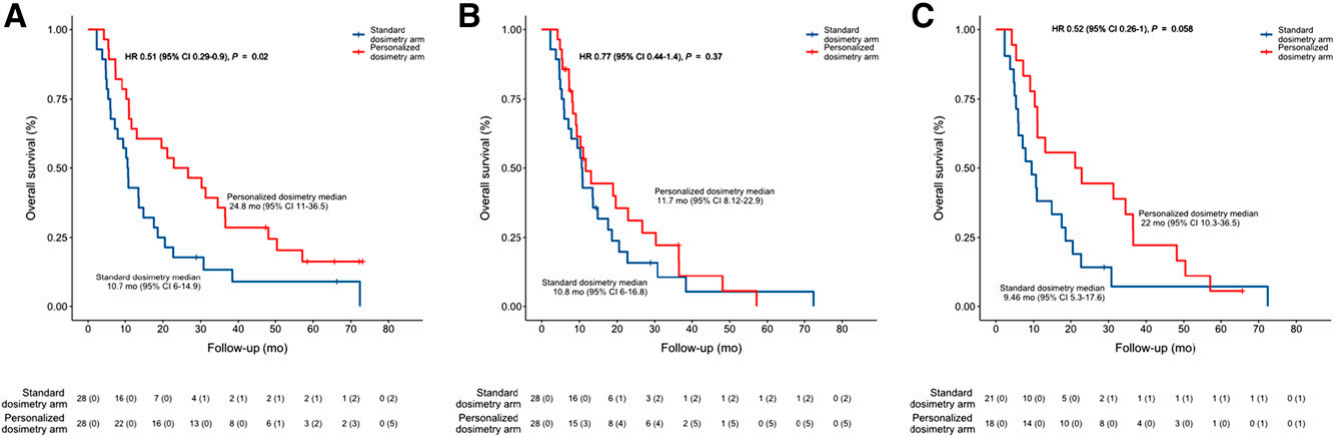
OS curves according to randomization for modified intent-to-treat population: median OS for global population (A), global population censored at time of surgery (B), and PVT patient subgroup (C).

**FIGURE 2.**
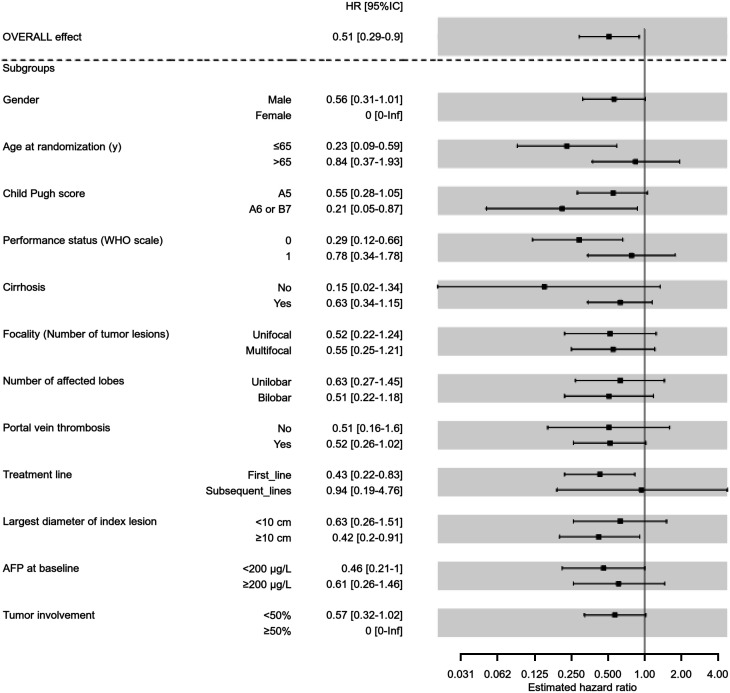
Forest plot analysis of HRs regarding treatment arms, for prespecified subgroups of interest in modified intent-to-treat population. AFP = α-fetoprotein; WHO = World Health Organization.

As shown in Figure 1, median OS was 24.8 mo (95% CI, 11–36.5 mo) in the PDA group versus 10.7 mo (95% CI, 6–14.9 mo) in the SDA group (HR, 0.51; 95% CI, 0.29–0.9; *P* = 0.020). The effect of personalized dosimetry was generally consistent across subgroups according to baseline characteristics (Fig. 2).

Censored at time of surgery, median OS was 11.7 mo (95% CI, 8.12–22.9 mo) in the PDA group versus 10.8 mo (95% CI, 6–16.8 mo) in the SDA group (HR, 0.77; 95% CI, 0.44–1.4; *P* = 0.37) (Fig. 1).

In patients with PVT, which was a patient subgroup of particular interest (*n* = 39), median OS was 22 mo (95% CI, 10.3–36.5 mo) in the PDA group versus 9.4 mo (95% CI, 5.3–17.6 mo) in the SDA group (HR, 0.52; 95% CI, 0.26–0.1; *P* = 0.058) (Fig. 1).

**FIGURE 3.**
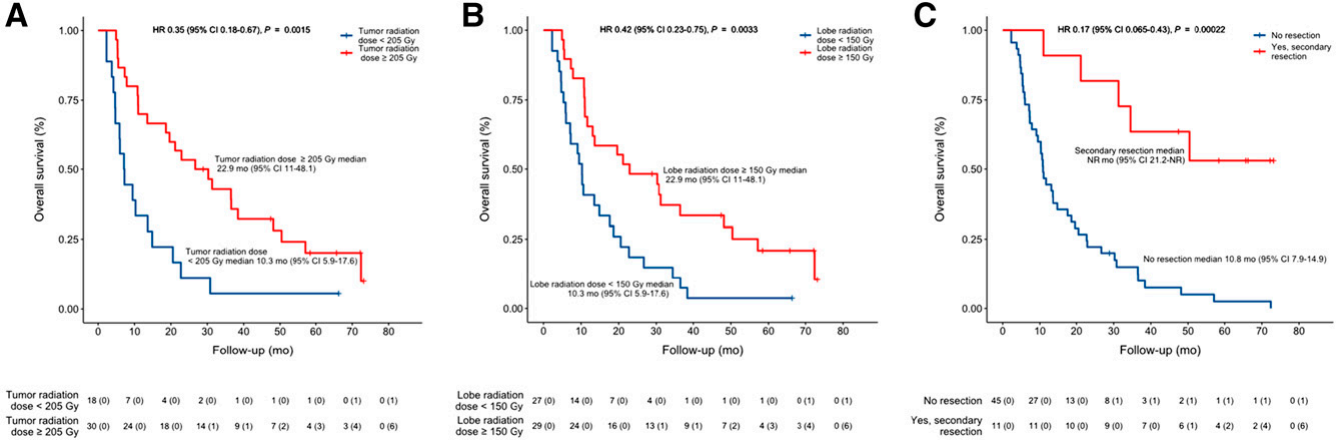
Median OS based on group of interest: TD (A), PLD (B), and secondary resection status (C). NR = not reached.

Concerning post hoc comparison (Fig. 3), median OS was 22.9 mo (95% CI, 11–48.1 mo) in patients with a TD of 205 Gy or higher versus 10.3 mo in those with a TD of less than 205 Gy (95% CI, 5.9–17.6 Gy; HR, 0.42; 95% CI, 0.22–0.81; *P* = 0.010). Median OS was 22.9 mo (95% CI, 11–48.1 mo) in patients with a PLD of 150 Gy or higher versus 10.3 mo in those with a PLD of less than 150 Gy (95% CI, 5.9–17.6 Gy; HR, 0.42; 95% CI, 0.23–0.75; *P* = 0.003). Lastly, median OS was not reached (95% CI, 21.2 mo to not reached) in patients who were secondarily resected (*n* = 11; 10 in the PDA group and 1 in the SDA group) versus 10.8 mo (95% CI, 7.9–14 mo) in those without secondary resection (*n* = 45) (HR, 0.17; 95% CI, 0.06–0.43; *P* < 0.001).

Estimated survival rates, by years, in the modified intent-to-treat population and subgroup population reported above have been presented in Table 2.

**TABLE 2.** OS Rates from 2 to 5 Years in Modified Intent-to-Treat Population

Parameter	2 y	3 y	5 y
PDA	50.0 (34.5–72.4)	35.7 (21.7–58.7)	16.4 (6.8–38.9)
SDA	17.8 (8–39.5)	13.3 (5–35.5)	8.9 (2.5–31.5)
PVT+ (PDA)	44.4 (26.5–74.5)	33.3 (17.3–64.1)	5.6 (0.8–37.3)
PVT+ (SDA)	14.2 (5–40.7)	7.1 (1.2–40.6)	7.1 (1.2–40.6)
TD ≥ 205 Gy	48.5 (34.1–68.9)	35.7 (22.4–56.8)	18.3 (8.5–39.1)
TD < 205 Gy	13.3 (3.6–48.4)	13.3 (3.6–48.4)	6.7 (1–44.3)
PLD ≥ 150 Gy	48.3 (33.1–70.4)	37.1 (22.9–60)	20.9 (9.8–44.2)
PLD < 150 Gy	18.5 (8.3–40.9)	11.1 (3.8–32.3)	3.7 (0.5–25.3)
Resected	81.8 (61.9–100)	63.6 (40.7–99.5)	53.0 (29.9–94)
Not resected	22.2 (12.8–38.4)	15 (7.8–30.5)	2.5 (0.3–17.1)

Data in parentheses are 95% CIs.

In multivariate analysis (Table 3), only 2 parameters were significantly associated with long-term OS: secondary resection (adjusted HR, 0.15; 95% CI, 0.06–0.43; adjusted *P* < 10^−3^) and bilobar disease (adjusted HR, 2.3; 95% CI, 1.24–4.37; adjusted *P* = 0.008).

**TABLE 3.** Multivariate Analysis of Factors Associated with OS

Factor	HR	*P*	Adjusted HR	Adjusted *P*
PDA vs. SDA	0.51 (0.29–0.9)	0.018	—	—
Resection after SIRT, yes vs. no	0.17 (0.06–0.43)	<10^−3^	0.15 (0.06–0.4)	<10^−3^
Sex, female vs. male	0.86 (0.27–2.77)	0.797	—	—
Age, >65 y vs. ≤65 y	1.13 (0.63–2)	0.684	—	—
Child Pugh score, A6 or B7 vs. A5	1.61 (0.84–3.1)	0.151	—	—
ECOG, 1 vs. 0	1.23 (0.69–2.19)	0.471	—	—
Cirrhosis, yes vs. no	2.4 (0.94–6.12)	0.059	—	—
Multifocal vs. unifocal	20.3 (1.13–3.65)	0.016	—	—
Bilobar vs. unilobar	2.1 (1.17–3.76)	0.011	2.33 (1.24–4.37)	0.008
PVT, yes vs. no	1.88 (0.98–3.63)	0.054	—	—
Treatment line, subsequent vs. first	1.09 (0.54–2.21)	0.808	—	—
Index lesion size, ≥10 cm vs. <10 cm	0.99 (0.56–1.76)	0.968	—	—
AFP, ≥200 μg/L vs. <200 μg/L	1.6 (0.91–2.83)	0.098	—	—
Tumor involvement, ≥50% vs. <50%	0.95 (0.29–3.07)	0.927	—	—
TD, ≥205 Gy vs. <205 Gy	0.42 (0.22–0.81)	0.007	—	—
PLD, ≥150 Gy vs. <150 Gy	0.42 (0.23–0.75)	0.002	—	—

ECOG = Eastern Cooperative Oncology Group; AFP = α-fetoprotein.

Data in parentheses are 95% CIs.

## DISCUSSION

After analysis of long-term 65.8-mo follow-up, improvement in median OS was shown to be sustained in the PDA group. The 22.9-mo (95% CI, 11–36.5 mo) median OS reached in the PDA group was observed in a population with severely advanced disease, including PVT involvement for 65% of them and a mean tumor size of 10.6 cm (7). These results compare favorably with results obtained in immunotherapy trials in which the reported median OS was 19.4 mo (95% CI, 11–36.5 mo) with atezolizumab plus bevacizumab (9) and 16.4 mo (95% CI, 14.1–19.5 mo) with durvalumab plus tremelimumab (10).

However, it must be mentioned that a direct comparison between SIRT studies and studies using systemic drugs turns out to be hazardous, especially on account of differences in the patient populations included. Indeed, in SIRT trials, PVT was shown to be more common (∼65% in DOSISPERE-01 (7) vs. only 26%–38% in immunotherapy trials (9*,*^10^)); SIRT patients did not exhibit any extrahepatic spread, whereas 53%–63% of immunotherapy-treated patients exhibited distant metastases (9*,*^10^); additionally, underlying cirrhosis characteristics and etiology differed (hepatitis B was reported in only 26% of SIRT-treated patients (7) vs. 31%–49% of immunotherapy-treated ones (9*,*^10^)).

Results for PVT patients deserve to be further highlighted, given that this patient population is of specific interest. Indeed, PVT patients were classified as advanced patients according to the Barcelona Clinic of Liver Cancer classification (2), similarly to patients with extrahepatic spread, despite portal vein invasion representing only a locoregional spread, which is thus accessible to SIRT (3*,*^11^*,*^12^), unlike distant metastasis. On the basis of this classification, the recommended treatment of PVT patients is systemic therapy rather than locoregional therapy such as SIRT (2). Although the statistically significant difference in OS for PVT patients was lost in this long-term analysis, this was most likely due to a lack of power, as this study was stopped by anticipation, whereas the trends are still striking, with a median OS of 22 mo (95% CI, 10.3–36.5 mo) in the PDA group versus 9.5 mo (95% CI, 5.3–17.6 mo) in the SDA group (HR, 0.52; 95% CI, 0.26–0.1; *P* = 0.058). Here, again, the median OS of 22 mo (95% CI, 10.3–36.5 mo) that was reached in the PDA group in PVT patients compares favorably with that obtained in immunotherapy-treated patients, for whom the median OS in the event of macrovascular invasion was 14.2 mo (95% CI, 11–19.4 mo) under atezolizumab plus bevacizumab (9) but was not reported for the durvalumab-plus-tremelimumab combination (10).

Regarding dosimetry parameters, the impact of TD on OS remains significant. Furthermore, this study revealed a significant impact exerted by PLD on OS (not reported in the first report). Indeed, median OS was 22.9 mo (95% CI, 11–48.1 mo) for patients receiving a PLD of 150 Gy or more versus 10.3 mo (95% CI, 5.9–17.6 mo) for those treated with a PLD of less than 150 Gy. Although TD and PLD are not independent prognostic indicators, this point is of particular interest for technical reasons when personalized dosimetry based on TD is difficult to perform. This can be the case given that tumor segmentation can turn out to be challenging in several instances (disease not well delineated, infiltrative disease, or multiple lesions), as when there is a large lesion with multiple feeders. In this situation, ^99m^Tc-macroaggregated albumin dosimetry would often require, to be accurate, 1 injection of ^99m^Tc-macroaggregated albumin for each feeder, separated by at least 24 h. In those situations, a PDA based on the PLD is doable, as for radiation segmentectomy (13*,*^14^), in this specific patient population exhibiting good liver function and at least 30% of hepatic reserve.

The analysis of OS rates from 2 to 5 y likewise discloses additional information of interest. For patients with poor features, namely those randomly assigned to SDA, receiving a TD of less than 205 Gy or a PLD of less than 150 Gy, or not downstaged to resection, OS rates were dramatically decreased, from 13% to 22% at 2 y and to less than 10% at 4 y. For patients with good features, besides those who were resected, OS rates were between 44% and 50% at 2 y and between 33% and 37% at 3 y. Only resected patients displayed an OS rate of more than 50% at 5 y.

The huge prognostic impact of secondary surgery on long-term OS in this population of patients with large lesions and often PVT, even with PDA, is highlighted by the loss of difference in median OS censored at the time of surgery between arms, as by the multivariate analysis. This result, the major impact of secondary resection to achieve prolonged long-term OS even with personalized dosimetry with large lesions and PVT, was not necessarily intuitive as it is not the case for small lesions. Indeed, in the LEGACY study (15), with 94% of lesions smaller than 5 cm (and 62% < 3 cm), OS was not driven by secondary surgery. Indeed, for patients who received SIRT as a unique treatment, OS was similar to that of patients with resection or transplantation (3-y OS rate of 86.6% without secondary surgery vs. 92.8% for patients with resection or transplantation) (15). The difference in the impact of surgery between these 2 kinds of populations can be explained by the fact that complete pathologic response is more frequently observed for small lesions, that is, in 67% of the patients of the LEGACY study who underwent resection or transplantation (14) versus only 10% for large lesions with often PVT in the DOSISPHERE-01 study (7). Furthermore, patients with large lesions and often PVT have a much higher risk of recurrence (median PFS was only 6 mo in the DOSISPHERE-01 study vs. not reached at 24 mo in the Legacy study (15)), then SIRT allowed to accurately evaluate the biological test of time, allowing surgery to be performed only on patients with a low risk of recurrence.

Two key messages arise from these observations. First, everything possible has to be done with SIRT to downstage patients to surgery, even including PVT patients, as it is the only way to achieve acceptable prolonged median OS rates, including an OS of more than 50% at 5 y. Such prolongation of median OS has recently been described after post-SIRT surgery performed for initially unresectable patients (16). In that study, including 18 patients who were accurately downstaged and then resected, 78% of whom presented with PVT, median OS was 61.8 mo (95% CI, 31.4 mo to not reached) (16). Thus, SIRT is most likely to optimize preparation and accurate selection of good PVT candidates who are eligible for surgery on account of its strong debulking effect (including portal vein complete response and revascularization) (7*,*^11^) and its ability to ensure contralateral liver hypertrophy, which is usually attained within 3–6 mo (17*,*^18^). In addition, biological tests performed in a timely manner permit exclusion of patients with early relapse after SIRT.

The second key message arising from our analysis is that even for patients with good features, excepting the resected ones, the OS rates were seen to decrease quite rapidly between 2 and 4 y, which is another strong argument to evaluate SIRT delivered in combination with immunotherapy in this patient population. Indeed, several arguments are in favor of combining SIRT with immunotherapy, including the strong debulking effect of SIRT in the treated area, even in the presence of large lesions (7). The systemic action of immunotherapy will be complementary to the local action of SIRT. Furthermore, a potential synergy between both approaches is awaited as SIRT is known to induce an immune response (19*,*^20^). Additional arguments for these combinations could be situations in which immunotherapy efficacy is likely diminished, such as in patients without viral hepatitis, in whom the HR was found to be 1.05 with atezolizumab plus bevacizumab versus sorafenib (9) but 0.58 for patients with viral hepatitis B. This may also apply to large lesions. Indeed, the response rate (modified RECIST) for the atezolizumab-plus-bevacizumab arm was significantly (*P* = 0.0097) lower in the presence of lesions larger than 5 cm than in the presence of lesions smaller than 5 cm, being 26.1% versus 40.9%, respectively (data calculation based on results presented in Fig. 1 (21)).

In addition to the rather small number of patients included, a major limitation of this study is that SIRT using personalized dosimetry was not randomized to this population’s standard treatment, consisting of atezolizumab plus bevacizumab or double immunotherapy. Therefore, drawing definite conclusions about the role of SIRT in this specific population is not possible. Thus, further randomized studies are warranted to better define this role.

## CONCLUSION

After a long-term follow-up period, a meaningful improvement in OS was sustained after personalized dosimetry. OS was dramatically improved for patients who were accurately downstaged toward resection and then resected, including most PVT patients. However, except for resected patients, the 5-y survival rates remain quite low. Randomized trials comparing SIRT with personalized dosimetry plus immunotherapy versus immunotherapy alone are now warranted in this specific patient population to better define the place of SIRT for this indication.

## DISCLOSURE

Etienne Garin reports receiving a grant, personal fees, and nonfinancial support from Boston Scientific during the conduct of the study. Lambros Tselikas reports personal fees from Boston Scientific, Sirtex, and GE Healthcare; grants from Terumo and the Bristol Myers Squibb Foundation; and nonfinancial support from GE Healthcare during the conduct of the study. Boris Guiu reports personal fees from Boston Scientific during the conduct of the study. Julien Edeline reports receiving a grant from Boston Scientific during the conduct of the study; personal fees from Boston Scientific, Bayer, Roche, Eisai, Merck Sharpe & Dohme, AstraZeneca, and Ipsen; grants and personal fees from Bristol Myers Squibb; and nonfinancial support from Amgen outside the submitted work. Thierry de Baere reports grants from Terumo and personal fees from Guerbet and Terumo during the conduct of the study. Antoine Hollebecque reports nonfinancial support from Servier, Incyte, and Lilly and personal fees from Amgen, Eisai, and Gritstone Oncology outside the submitted work. Helen Regnault reports personal fees from Boston Scientific outside the submitted work. Xavier Palard, Boris Campillo-Gimenez, and Yan Rolland report receiving a grant from Boston Scientific during the conduct of the study. No other potential conflict of interest relevant to this article was reported.

## Notes

KEY POINTS **QUESTION:** Does long-term analysis maintain substantial OS improvement for locally advanced hepatocellular carcinoma patients treated with SIRT using personalized dosimetry? **PERTINENT FINDINGS:** In this randomized study comparing personalized dosimetry versus a standard dosimetry, OS improvement was maintained during long-term analysis of 5 y. This is particularly true for patients accurately downstaged to resection, whose OS rate at 5 y was higher than 50%. **IMPLICATIONS FOR PATIENT CARE:** SIRT must be used with personalized dosimetry for locally advanced hepatocellular carcinoma, and SIRT with personalized dosimetry may become the standard care for well-selected patients with advanced hepatocellular carcinoma.
